# Novel *Rht-1* dwarfing genes: tools for wheat breeding and dissecting the function of DELLA proteins

**DOI:** 10.1093/jxb/erw509

**Published:** 2017-02-15

**Authors:** Stephen G. Thomas

**Affiliations:** 1 Plant Biology and Crop Science Department, Rothamsted Research, Harpenden, Hertfordshire, AL5 2JQ, UK

**Keywords:** DELLA proteins, dormancy, gibberellins, Green Revolution, preharvest sprouting, *Rht-1* dwarfing mutations, stem height, wheat (*Triticum aestivum*).

*Reduced height-1* (*Rht-1*) dwarfing alleles have provided an essential breeding tool for increasing wheat grain yields and providing lodging resistance under high inputs. In this issue (pages 443–455), Van De Velde *et al.* demonstrate the potential of novel *Rht-1* alleles for allowing more flexible control of stature and preharvest sprouting resistance in wheat breeding programmes. On a fundamental level, these alleles provide an important opportunity to uncover the signalling events that are responsible for improving these traits.

During the Green Revolution, the benefits of intensive agronomic practices to increase wheat grain yields could only be fully achieved when combined with varieties containing *Reduced height* (*Rht*) dwarfing genes (Hedden, 2003). The beneficial effects of these dwarfing alleles on grain yields are twofold: first, they prevent excessive stem elongation in response to high nitrogen fertilizer regimes that are prone to make the crop susceptible to environmental damage through lodging. Second, they allow a higher proportion of photosynthate to be partitioned to the grain by increasing the number of grains within the spikelets of the spike. The most widely utilized *Rht* dwarfing genes in worldwide wheat breeding programmes are those containing lesions at the *Rht-1* locus. These include the *Rht-B1b* and *Rht-D1b* semi-dwarfing alleles which, at around the turn of the last century, were estimated to be present in over 70% of wheat cultivated worldwide (Evans, 1998). Remarkably, both of these *Rht-1* homoeo-alleles originated from the same Japanese variety, Norin-10, and were successfully exploited in US wheat breeding programmes in the 1950s (Hedden, 2003; Wilhelm *et al.*, 2013).

Gibberellins (GAs) are plant hormones that promote many aspects of vegetative and reproductive development, including stem elongation and germination (Sponsel, 2016). The central regulators of the GA signalling pathway are nuclear-localized DELLA proteins (DELLAs) that act to repress GA-responsive growth through their physical association with transcription factors and other down-stream components (Thomas *et al.*, 2016). Bioactive GAs relieve the DELLA growth repression by targeting their rapid degradation through a GID1–GA receptor-mediated signalling pathway (Nelson and Steber, 2016). The *Rht-1* dwarfing alleles are known to reduce stem extension by causing partial insensitivity to GAs (Gale and Marshall, 1973). This altered response is caused by mutations in the homoeologous DELLA genes *Rht-B1* and *Rht-D1* (Peng *et al.*, 1999; Pearce *et al.*, 2011).

DELLAs are members of the GRAS family of transcriptional regulators (Thomas *et al.*, 2016), containing two distinct domains: an N-terminal regulatory domain and a C-terminal functional GRAS domain (Box 1). The N-terminal domain is required for binding the GID1–GA receptor complex, a process which ultimately triggers DELLA degradation and promotes GA-responsive growth. The agronomically important *Rht-B1b* and *Rht-D1b* semi-dwarfing alleles contain mutations that introduce premature stop codons in the region of these genes encoding the N-terminal GID1–GA binding domain (Peng *et al.*, 1999; Pearce *et al.*, 2011; Box 1). It is believed that the effect of these mutations is to produce an N-terminally truncated protein which cannot be bound by the GID1–GA receptor, therefore resisting GA-mediated degradation and acting to constitutively repress GA-responsive growth and development. The severe dwarfing allele, *Rht-B1c*, also contains a lesion in the N-terminal coding region, which is predicted to have the same effect on perturbing GA signalling. However, in this case, the increased stability of RHT-B1C is due to a 30-amino acid insertion within the GID1–GA binding domain (Pearce *et al.*, 2011; Wu *et al.*, 2011). Although conclusive biochemical evidence is lacking, the current consensus of opinion regarding the milder GA-insensitive phenotype observed in *Rht-B1b* and *Rht-D1b* compared to *Rht-B1c* is due to a lower level of accumulation of the N-terminally truncated proteins produced by a process of translational reinitiation (Peng *et al.*, 1999).

**Box 1. *Rht-1* dwarfing mutations**

The upper panel shows an amino acid alignment of a region of the N-terminal regulatory domains of the wheat, rice and Arabidopsis DELLAs. The blue bars and asterisks below the alignment indicate conserved residues required for binding to the GID1–GA receptor (Murase et al., 2008), targeting them for degradation and relieving growth repression. The positions of mutations are shown in *Rht-B1c* (purple triangle indicating the site of a 30 amino acid insertion) and *Rht-B1b* (green triangle indicating the introduction of a premature stop codon; Q64*). Translational reinitiation (potential sites are indicated by a green bar) is thought to produce an N-terminally truncated RHT-B1B protein. The current model suggests that RHT-B1C and RHTB1B constitutively repress GA-responsive growth because they do not bind to the GID1–GA complex and are therefore not degraded in response to the GA signal. The lower panel shows a schematic diagram illustrating conserved domains within RHT-B1 and the predicted effect of *Rht-1* dwarfing mutations on the encoded proteins. The positions of amino acid substitutions caused by the ovg missense mutations are indicated within the GRAS domain (vertical blue lines) (Chandler and Harding, 2013; Van de Velde et al. 2017).

**Figure:**
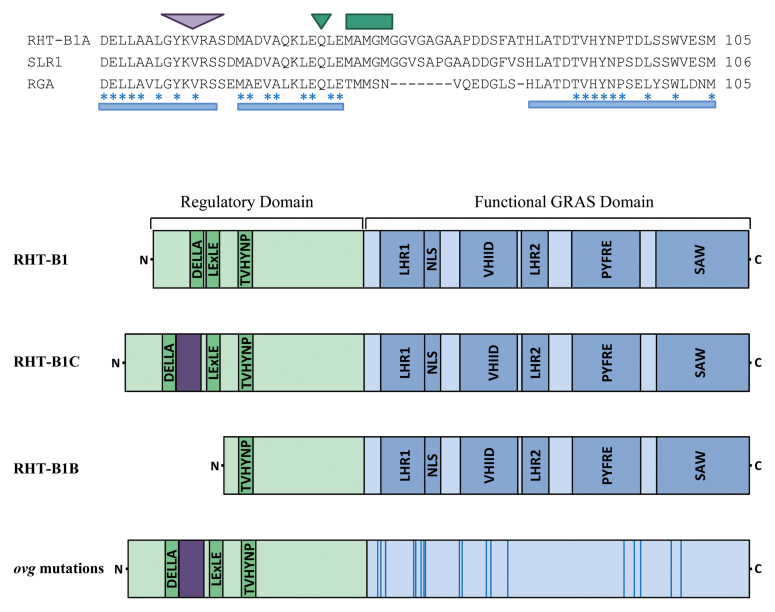

## Novel *Rht-1* dwarfing alleles

Despite the success of *Rht-1* dwarfing alleles in controlling wheat stature and grain yields, the current range of genetic and phenotypic diversity is limited. An elegant screen performed by Chandler and Harding (2013) has greatly extended this diversity (Box 2). In this study they identified taller suppressor lines, designated *overgrowth* (*ovg*) alleles, following mutagenesis of a severely dwarfed line containing *Rht-B1c*. A total of 35 intragenic *Rht-B1c* suppressor alleles were identified, the majority of mutations resulting in amino acid substitutions within conserved regions of the functional GRAS domain (Chandler and Harding, 2013; Van De Velde *et al.*, 2017; Box 1). As well as providing alleles that have the potential to allow breeders to precisely control stature over a wider range, the nature of these mutations provides us with the tools for understanding how DELLAs act to control diverse developmental processes. These studies demonstrate the exciting potential of these alleles on both of these levels.

**Box 2. Wheat overgrowth mutants**

The wheat overgrowth mutants (var. Maringa) were identified by mutagenizing the severe dwarf line *Rht-B1c* and screening for M2-suppressor lines that displayed increased leaf elongation or final height (Chandler and Harding, 2013). The screen resulted in the identification of many new *Rht-B1* dwarfing alleles that can be used for more precise control of wheat stem height. Illustrating this, the image shows two ovg mutants (*Rht-B1c.32* and *Rht-B1c.3*) compared to wild-type *Rht-B1a* (tall control), and the classical *Rht-1* dwarfing lines *Rht-B1b* (semi dwarf) and *Rht-B1c* (extreme dwarf). Picture courtesy of Karel Van De Velde.

**Figure:**
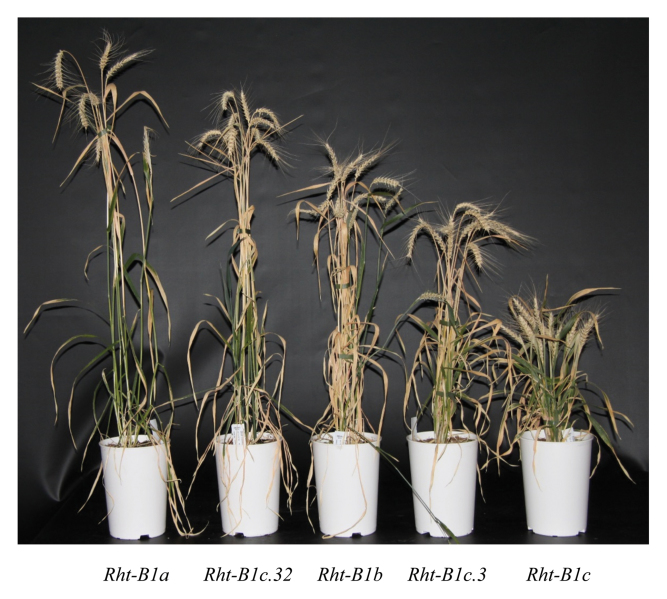

To establish whether the *ovg* alleles represented useful alternatives to the classical dwarfing gene, *Rht-B1b*, Van De Velde *et al.* (2017) selected fourteen for further phenotypic characterization. Based on stem height these were classified as semi-dwarfing or tall depending on whether they were shorter or taller than *Rht-B1b*, respectively (Box 2). This allowed the selection of three alleles (two semi-dwarfing and one tall) whose agronomic potential was established following introgression into several elite spring wheat cultivars that have markedly different continental growing environments. Highly consistent effects on stem length and other architectural characteristics were observed in the presence of the *ovg* alleles. Importantly, no obvious detrimental effects on grain yields were observed under field conditions. The stability that they exert on these crop traits in different genetic backgrounds and under diverse environments clearly demonstrates their potential in wheat breeding programmes.

## *Rht-1* alleles with differential effects on GA-responsive traits

During the past decade there have been many studies demonstrating that DELLAs interact with a multitude of different classes of proteins to repress aspects of GA-responsive growth (Thomas *et al.*, 2016). The regulatory mechanisms that these associations affect also differ depending on the interaction partner. Despite the large variety of DELLA partners and the diverse regulatory mechanisms involved, the majority of these studies have identified the GRAS domain as responsible for mediating these associations and exerting control. This is in agreement with the position of *ovg* mutations causing amino acid substitutions, which were identified solely within the region encoding the GRAS domain of RHT-B1C (Chandler and Harding, 2013; Van De Velde *et al.*, 2017). Van De Velde *et al.* (2017) have used the recently resolved crystal structure of the rice GRAS protein OsSCL7 to model the effect of *ovg* mutations (Li *et al.*, 2016). Many of these substitutions are suggested to occur within the interior of the DELLA protein, potentially affecting interacting residues (Van De Velde *et al.*, 2017). It is tempting to speculate that the impact of these is to alter the 3D structure and perturb interactions with down-stream GA signalling components. To establish whether this is the case, it will first be necessary to identify RHT-1-interacting partners and then determine the impact of the *ovg* mutations on these interactions.

Based on our current understanding of how *Rht-1* dwarfing genes cause GA insensitivity it might be reasonable to expect that the impact of a particular allele has an equivalent effect across different developmental processes controlled by GAs. However, there is evidence that this is not the case: a recent study of *Rht-1* near-isogenic lines has indicated that those alleles containing premature stop codons, including the severe dwarf *Rht-D1c*, do not dramatically affect grain dormancy whereas *Rht-B1c* enhances it strongly (Gooding *et al.*, 2012). The characterization of germination responses in the *ovg* mutants derived from *Rht-B1c* clearly confirms these differences between alleles, with the majority of them producing a level of dormancy that is consistent with their effect on stature (Van De Velde *et al.*, 2017). In contrast, this correlation was not observed in the *Rht-B1b* semi-dwarf controls, in which dormancy was unaffected. This suggests an uncoupling of GA-regulated developmental responses that are controlled by the RHT-1 repressors lacking 70 amino acids of the N-terminus (Van De Velde *et al.*, 2017). It is interesting to note that there is evidence supporting a functional role for the N-terminus of the rice DELLA SLR1 (Hirano *et al.*, 2012). If the DELLA N-terminus does have a functional role, what is perhaps surprising is the absence of *ovg* missense mutations in this region. A plausible explanation for this could be the design of the original screen, which involved identifying enhanced leaf or stem elongation (Chandler and Harding, 2013). If feasible, a similar screen focusing on germination responses may deliver alternative intragenic suppressor alleles. Nevertheless, from the perspective of crop improvement, it is clear that the enhanced dormancy observed in the *ovg* mutants provides the potential to increase resistance to preharvest sprouting beyond that obtained with the currently deployed *Rht-1* alleles.

## Wheat: a new model plant

Attempts aimed at manipulating specific signalling pathways to improve traits in bread wheat have been severely hindered by its hexaploid nature and the lack of a reference genome sequence. The recent availability of a near-complete genome sequence now raises exciting possibilities for quickly identifying genetic elements underlying these traits (Borrill *et al.*, 2015). An obvious problem with the hexaploid genome is that the presence of functionally redundant homoeologues impedes the identification of recessive loss-of-function alleles, probably obscuring genetic diversity that can be exploited by wheat breeders. The recent development of robust reverse genetics platforms for functional genomics in wheat, including TILLInG populations (King *et al.*, 2015) and CRISPR/Cas-based mutagenesis (Wang *et al.*, 2014), raises the exciting prospect of unlocking this genetic diversity. An advantage of polyploid genomes is that mutagenesis events can be chemically induced at much higher frequencies than those achieved in diploid species, allowing the generation of increased genetic diversity (Parry *et al.*, 2009). The unparalleled collection of DELLA mutations identified by Chandler and Harding (2013) clearly emphasizes these benefits. It also illustrates the power of novel screening strategies for generating improved genetic diversity that is unavailable with conventional breeding approaches.
